# Morphological and molecular characterization of *Sarcocystis wenzeli* in chickens (*Gallus gallus*) in China

**DOI:** 10.1186/s13071-020-04390-x

**Published:** 2020-10-14

**Authors:** Jing Pan, Chunli Ma, Zhumei Huang, Yulong Ye, Hongxia Zeng, Shuangsheng Deng, Junjie Hu, Jianping Tao

**Affiliations:** 1 School of Biological Sciences, Yunnan University, Kunming, 650091 China; 2 School of Ecology and Environmental Sciences and Yunnan Key Laboratory for Plateau Mountain Ecology and Restoration of Degraded Environments, Yunnan University, Kunming, 650091 China; 3 College of Veterinary Medicine, Yangzhou University, Yangzhou, 225009 China

**Keywords:** *Sarcocystis wenzeli*, *Gallus gallus*, Morphological and molecular characterization

## Abstract

**Background:**

There has been considerable confusion concerning the number and classification of *Sarcocystis* spp. in chickens. Scarce nucleotide data of *Sarcocystis* spp. from chickens are provided in GenBank. The study aimed to investigate the morphological and molecular characteristics of *Sarcocystis* spp. found in chickens in China.

**Methods:**

Tissues from 33 chickens were collected in 2019. Sarcocysts were observed using light (LM) and transmission electron microscopy (TEM). Individual sarcocysts from different chickens were selected for DNA extraction, and five loci, *18S* rDNA, *28S* rDNA, ITS1 region, the mitochondrial *cox*1 gene and the apicoplastic *rpoB* gene, were amplified from each sarcocyst, sequenced and analyzed.

**Results:**

Only *S*. *wenzeli* was found in 14 of 33 (42.4%) chickens. Under LM, the sarcocysts were microscopic and exhibited palisade-like villar protrusions measuring 1.5–2.8 μm. Ultrastructurally, the sarcocyst wall contained numerous stubby hill-like villar protrusions. The protrusions included scattered microtubules, which extended from the tips of the protrusions into the ground substance. The five loci were successfully sequenced and the sequences deposited in GenBank. At *18S* rDNA, ITS1 and *cox*1, the most similar sequences in GenBank were those of *Sarcocystis* sp. obtained from the brains of chickens, i.e. 99.9–100%, 98.1–98.5% and 99.3% identity, respectively. The five loci (*18S* rDNA, *28S* rDNA, ITS1, *cox*1 and *rpoB*) showed different levels of interspecific sequence similarity with other closely related species of *Sarcocystis* (e.g. 99.8%, 99.0–99.2%, 89.3–89.7%, 98.5%, and 97.5%, respectively, with *S*. *anasi*). Phylogenetic analysis based on four of the loci (*18S* rDNA, *cox*1, *rpoB* and ITS1) revealed that *S*. *wenzeli* formed an independent clade with *Sarcocystis* spp. that utilize geese or ducks as intermediate hosts and canines as the known or presumed definitive host.

**Conclusions:**

To our knowledge, the sequences of *28S* rDNA and *rpoB* reported here constitute the first records of genetic markers of *Sarcocystis* spp. in chickens. Based on molecular analysis, *S*. *wenzeli* might be responsible for the neurological disease in chickens, and ITS1 and *rpoB* are more suitable for discriminating it from closely related *Sarcocystis* spp. Phylogenetic analysis revealed that *S*. *wenzeli* presents a close relationship with *Sarcocystis* spp. in geese or ducks. 
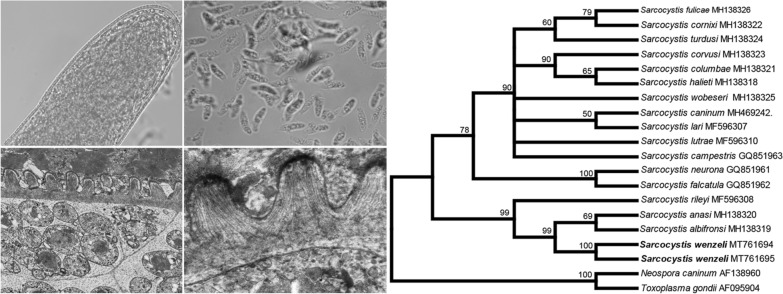

## Background

Species of the genus *Sarcocystis* exhibit an obligate two-host life-cycle, with sexual development in the small intestine of the definitive host and asexual development in different tissues of the intermediate host, which are usually herbivores. To date, three species of *Sarcocystis* with chickens (*Gallus gallus*) as the intermediate host have been named: *S*. *horvathi* Rátz, 1908 [1], *S*. *gallinarum* Krause and Goranoff [2] and *S*. *wenzeli* (Wenzel et al.) Odening 1997 [3, 4]. However, there has been considerable confusion concerning the number and classification of species of *Sarcocystis* in chickens owing to the imperfection of the original description [5].

The correct identification of *Sarcocystis* species that might infect chickens is crucial for sarcocystosis control and prevention. The ultrastructure of sarcocysts is traditionally a reliable characteristic for identifying different *Sarcocystis* species in a given host. Currently, PCR assays and sequencing procedures are considered much more practical, accurate, and reliable methods for the delineation and identification of *Sarcocystis* species than traditional methods based on morphological characteristics [6, 7]. However, there are only one *18S* rDNA sequence (783 bp), one ITS1 region (ITS1) sequence (923 bp) and one mitochondrial *cox*1 gene sequence (547 bp) of *Sarcocystis* sp. in chickens currently deposited in GenBank. All these nucleotide sequences were obtained from *Sarcocystis* in chickens associated with neurological lesions in Brazil, and the parasite was closely related to *S*. *anasi* and *S*. *albifronsi* [8]. Nevertheless, *Sarcocystis* sp, found by these authors was not identified to the species level.

Therefore, the aims of the present study were: (i) to obtain data on the prevalence of *Sarcocysits* in chickens using morphological characteristics; to (ii) sequence and analyze the near-complete *18S* rDNA, *28S* rDNA, ITS1, *cox*1 and apicoplastic rpoB gene (*rpoB*) of *Sarcocystis* species found in chickens in order to augment the species descriptions; and (iii) to investigate phylogenetic relationships of *Sarcocystis* species in chickens with known species of fowl-infecting *Sarcocystis* spp. using *18S* rDNA, *cox*1, *rpoB* and ITS1 sequences.

## Methods

### Morphological examination of sarcocysts from chickens

In total, tissues from 33 chickens were collected from Jiaojiaqing village, Shizong County, Yunnan Province, located in southwestern China, in July and December 2019. These chickens were free ranging and were raised by the local peasants. From each chicken, fresh tissue samples of the skeletal muscles and heart were examined for sarcocysts. In the laboratory, 20 pieces of 3 mm muscle from each collected sample were pressed and squeezed between two glass slides and inspected using a stereomicroscope. Individual sarcocysts were extracted and isolated from muscle fibers using dissection needles and processed for light (LM) and transmission electron microscopy (TEM) and DNA analysis. For TEM, four sarcocysts (two from chicken no. 4 and two from chicken no. 10) were fixed in 2.5% glutaraldehyde in cacodylate buffer (0.1 M, pH 7.4) at 4 °C and post-fixed in 1% osmium tetroxide in the same buffer, then dehydrated in a graded alcohol series and embedded in an epon-alaldite mixture. Ultrathin sections were stained with uranyl acetate and lead citrate and then examined using a JEM100-CX transmission electron microscope (JEOL Ltd., Tokyo, Japan) at 80 kV. For DNA isolation, individual cysts were stored in sterile water at −20 °C prior to processing.

### DNA isolation, PCR amplification, cloning and sequence analysis

For DNA analysis, 5 individual sarcocysts isolated from different chickens were subjected to genomic DNA extraction using the TIANamp Genomic DNA Kit (Tiangen Biotech Ltd., Beijing, China) according to the manufacturer’s instructions. The *Sarcocystis* species were characterized at 5 loci within the *18S* rDNA, *28S* rDNA, ITS1, *cox*1 and *rpoB* genes. The near-complete *18S* rDNA gene was amplified with the primer pair S1/SarDR [9, 10]; the near-full-length *28S* rDNA gene was amplified with the primer sets KL1/KL3, KL4/KL5b, and KL6/KL2 [11]; the complete ITS1 region was amplified with the primer pair P-ITSF/P-ITSR [10]; the partial *cox*1 gene was amplified with the primer pair SF1/COIRm [6]; and the partial *rpoB* fragment was amplified with the primer pair rpoBF2 (5'-ATT TTT GTG GAT ATG ATT TTG AAG ATG C-3') and rpoBR2 (5'-AGT TTA GAT CCA GTT CTA CCG-3'), designed using OLIGO 7.60 (Molecular Biology Insights, Inc., West Cascade, USA) based on highly conserved regions of the *rpoB* sequences of *Toxoplasma gondii*, *Neospora caninum*, and *Sarcocystis* spp. deposited on GenBank. The PCR products were purified, cloned, sequenced, and assembled using the methods described in a previous report [12].

Phylogenetic analyses were conducted separately for the *18S* rDNA, *cox*1, *rpoB* and ITS1 sequences using MEGA X software [13]. The selected sequences of the four loci of *Sarcocystis* spp. from various hosts were downloaded from GenBank, respectively, and aligned with the ClustalW program integrated in MEGA X applying a gap opening penalty of 10/10 and a gap extension penalty of 0.1/0.2 as pairwise and multiple alignment parameters, respectively. The alignment was subsequently checked visually; some sequences were truncated at both ends, so all sequences started and ended at the same nucleotide positions. The maximum parsimony (MP) trees were generated with a tree-bisection-regrafting (TBR) algorithm. The reliability of the MP phylograms was tested with the bootstrap method using 1000 replications.

In the case of *18S* rDNA, the final alignment comprised a total of 29 nucleotide sequences from 26 taxa and 2100 aligned positions, and *Besnoitia besnoiti* (GenBank: DQ227418), *N*. *caninum* (GenBank: U16159) and *T*. *gondii* (GenBank: U03070) were chosen as outgroups to root the tree. At *cox*1, the final alignment comprised 28 *cox*1 nucleotide sequences from 28 species and 1020 aligned positions with no gaps, and *T*. *gondii* (GenBank: JX473253), *Hammondia triffittae* (GenBank: JX473247) and *B*. *besnoiti* (GenBank: XM029362743) were used as outgroup species. At *rpoB*, the final alignment comprised 20 *rpoB* nucleotide sequences from 19 species and 694 aligned positions with no gaps, and *T*. *gondii* (GenBank: AF095904) and *N. aninum* (GenBank: AF138960) were used as outgroup species to root the tree. At ITS1, the final alignment comprised 25 ITS1 nucleotide sequences from 21 species and 1206 aligned positions, and *B*. *tarandi* (GenBank: MH217579) and *N*. *caninum* (GenBank: U16159) were chosen as outgroups.

## Results

### LM and TEM examination of *S*. *wenzeli* sarcocysts

**Fig. 1:**
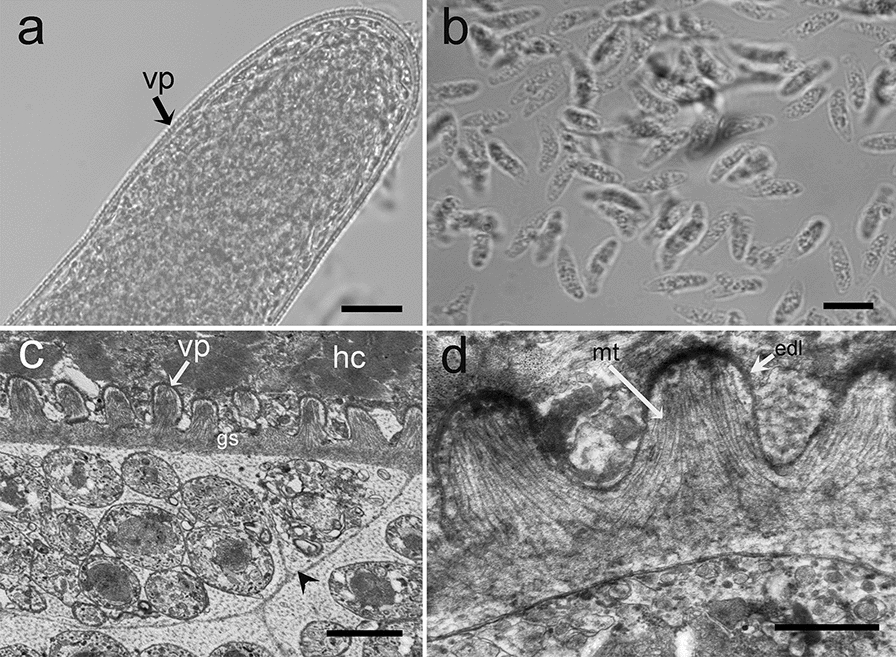
Morphological characteristics of *Sarcocystis wenzeli* isolated from skeletal muscle in chickens. **a** LM micrograph of a sarcocyst (unstained). Note the short palisade-like villar protrusions (vp). **b** LM micrograph of bradyzoites (unstained). Note the lancet-shaped bradyzoites. **c** TEM micrograph of a sarcocyst. Note the stubby hill-like villar protrusions (vp), ground substance (gs), septa (arrowhead), and host cell (hc). **d** TEM micrograph of a sarcocyst. Note the electron-dense layer (edl) and the microtubules (mt). *Scale-bars*: **a**, 20 μm; **b**, 10 μm; **c**, 2 μm; **d**, 1 μm

Only sarcocysts resembling those of *S*. *wenzeli* were found in 14 of 33 (42.4%) chickens. Sarcocysts were found in skeletal muscles but not in the heart. Using LM, the sarcocysts of the parasite were observed to be microscopic, measuring 381–3585 × 48–154 μm (*n* = 30). The sarcocyst wall exhibited numerous short palisade-like villar protrusions measuring 1.5–2.8 μm (*n* = 40) in length (Fig. 1a). The cysts were septate, and their interior compartments were filled with lancet-shaped bradyzoites measuring 9.2–12.6 × 1.5–3.5 μm (*n* = 40) (Fig. 1b).

Four sarcocysts from both chickens were examined using TEM, all of which appeared to have walls that were ultrastructurally similar and closely resembled the “type 9k” cyst wall. The sarcocyst wall contained numerous stubby hill-like villar protrusions that were up to 1.2 μm long and 1.0 μm wide and were lined with an electron-dense layer that appeared thicker at the tips of the protrusions (Fig. 1c). Within the protrusions, there were numerous scattered fine, electron-dense granules and scattered microtubules. The microtubules extended from the tips of the protrusions into the ground substance, where they crossed microtubules originating from neighboring protrusions (Fig. 1d). The protrusions were spaced at intervals of 0.3–1.1 μm from each other. Small invaginations of the primary wall were present on the lateral aspect of the protrusions and in the spaces between protrusions. The layer of ground substance beneath the protrusions was 0.3–0.4 μm in thickness; septa were evident within the cysts (Fig. 1c).

### Molecular analysis

Genomic DNA was extracted from the 5 individual sarcocysts of *S*. *wenzeli* isolated from different chickens, and *18S* rDNA, *28S* rDNA, ITS1, *cox*1 and *rpoB* were successfully amplified using their DNA as templates. The five *18S* rDNA sequences of *S*. *wenzeli* were 1747 bp in length and shared an identity of 99.8–100% (average 99.8% identity). Therefore, only 4 sequences (GenBank: MT756990-MT756993) were submitted to GenBank. The most similar sequence in GenBank was that of *Sarcocystis* sp. isolate Chicken-2016-DF-BR (MN845627) from chicken (99.9–100% identity, average 99.9% identity), followed by *S*. *anasi* (EU553477) from mallard duck (*Anas platyrhynchos*) (99.8% identity), *S*. *albifronsi* (EU502868) from white-fronted goose (*Anser albifons*) (99.7% identity) and *S*. *rileyi* (KJ396583) from common eider (*Somateria mollissima*) (99.5% identity).

The five *28S* rDNA sequences of *S*. *wenzeli* were 3279 bp in length and shared 99.7–100% identity (average 99.9% identity). Therefore, only 4 sequences (GenBank: MT756986-MT756989) were deposited in GenBank. The most similar sequence in GenBank was that of *S*. *albifronsi* (EF079885) (99.3–99.1% identity, average 99.2% identity), followed by *Sarcocystis* sp. (MH898978) from Temminck’s stint (*Calidris temminckii*) (98.9–99.2% identity, average 99.1% identity), *S*. *anasi* (EF079887) (99.0–99.2% identity, average 99.1% identity), *S*. *cornixi* (EU553480) from hooded crow (*Corvus cornix*) (98.6–98.9% identity, average 98.8% identity) and *S*. *rileyi* (GU188426) (98.3–98.5% identity, average 98.4% identity).

The five *cox*1 sequences of *S*. *wenzeli* were 1142 bp in length and were completely identical; therefore, only 1 sequence (MT761700) was submitted to GenBank. The most similar sequence in GenBank was that of *Sarcocystis* sp. isolate Chicken-2016-DF-BR (MN848337) from chicken (99.3% identity), followed by *S*. *albifronsi* (MH138310) (98.6% identity), *S*. *anasi* (MH138311) (98.5% identity) and *S*. *rileyi* (KJ396582) (96.4% identity).

The five ITS1 sequences (MT756994-MT756998) of *S*. *wenzeli* were 1186–1187 bp in length and shared 99.0–99.9% identity, with an average identity of 99.4%. The most similar sequence was that of *Sarcocystis* sp. isolate Chicken-2016-DF-BR (MN846302) from chicken (98.1–98.5% identity, average 98.3% identity), followed by *S*. *anasi* (JF520779) (89.3–89.7% identity, average 89.5% identity) and *S*. *rileyi* (KJ396584) (78.8–79.0% identity, average 78.9% identity).

The five *rpoB* sequences of *S*. *wenzeli* were 844 bp in length and shared 98.9–100% identity, with an average identity of 99.3%, so only 2 sequences (MT761694 and MT761695) were submitted to GenBank. The most similar sequence in GenBank was that of *S*. *anasi* (MH138320) (97.5% identity), followed by those of *S*. *albifronsi* (MH138319) (97.4% identity) and *S*. *rileyi* (MF596308) (95.9% identity).

**Fig. 2:**
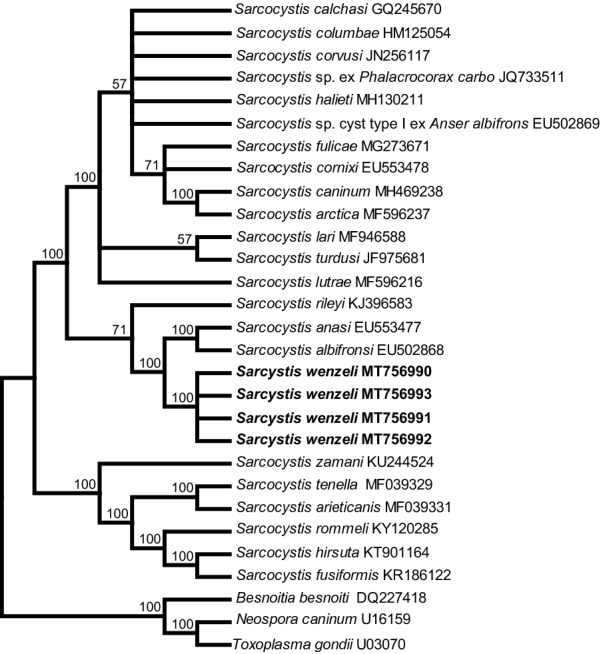
Phylogenetic tree of selected members of the family Sarcocystidae based on *18S* rDNA sequences, inferred using the maximum parsimony (MP) method with the tree bisection-regrafting algorithm (TBR). The values between the branches represent the percent bootstrap value per 1000 replicates, and values below 50% are not shown. The GenBank accession numbers of all the sequences included in the analysis are given after the taxon names. The four new sequences of *Sarcocystis wenzeli* (MT756990-MT756993) are shown in boldface

**Fig. 3:**
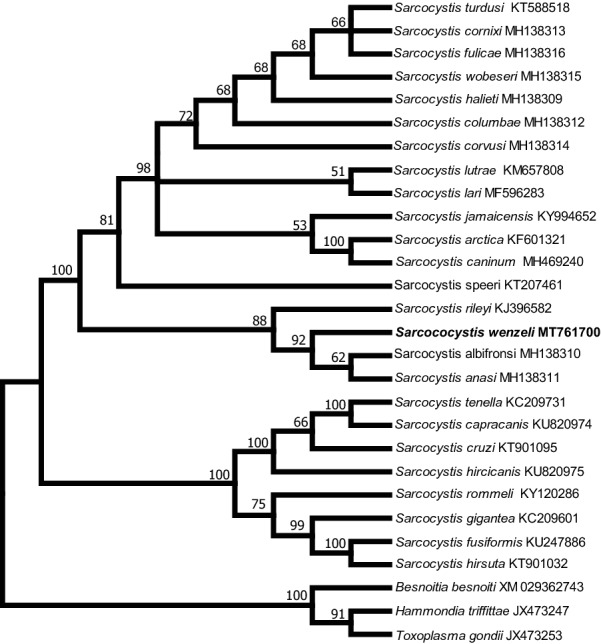
Phylogenetic tree of selected members of the family Sarcocystidae based on mitochondrial *cox*1 sequences, inferred using the maximum parsimony (MP) method with the tree bisection-regrafting algorithm (TBR). The values between the branches represent the percent bootstrap value per 1000 replicates, and values below 50% are not shown. The GenBank accession numbers of all the sequences included in the analysis are given after the taxon names. The new sequence of *Sarcocystis wenzeli* (MT761700) is shown in boldface

**Fig. 4:**
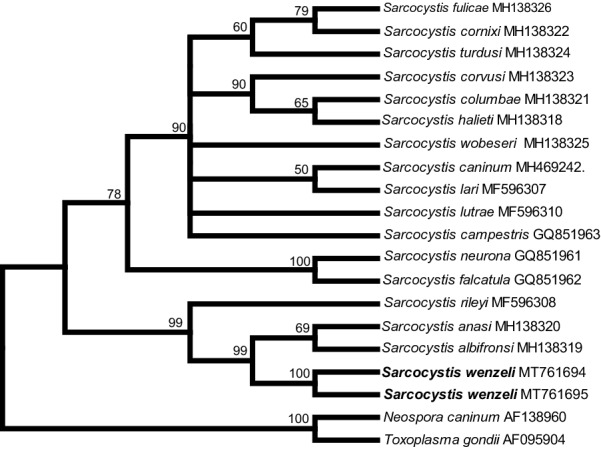
Phylogenetic tree of selected members of the family Sarcocystidae based on apicoplastic *rpoB* sequences, inferred using the maximum parsimony (MP) method with the tree bisection-regrafting algorithm (TBR). The values between the branches represent the percent bootstrap value per 1000 replicates, and values below 50% are not shown. The GenBank accession numbers of all the sequences included in the analysis are given after the taxon names. The two new sequences of *Sarcocystis wenzeli* (MT761694 and MT761695) are shown in boldface

**Fig. 5:**
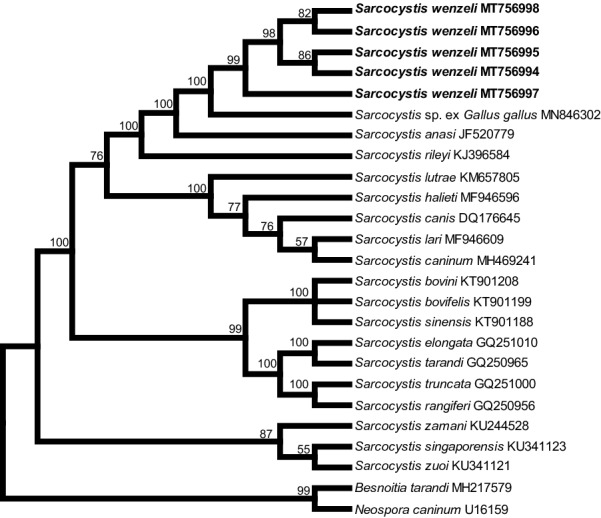
Phylogenetic tree of selected members of the family Sarcocystidae based on ITS1 sequences, inferred using the maximum parsimony (MP) method with the tree bisection-regrafting algorithm (TBR). The values between the branches represent the percent bootstrap value per 1000 replicates. The GenBank accession numbers of all the sequences included in the analysis are given after the taxon names. The five new sequences of *Sarcocystis wenzeli* (MT756994-MT756998) are shown in boldface

Although the newly obtained *18S* rDNA, *cox*1 and ITS1 sequences had the highest similarity with homologous sequences of *Sarcocystis* sp. isolate Chicken-2016-DF-BR, only the ITS1 sequence of this isolate was included in the phylogenetic analysis because sequences of the other two loci of the isolate were shorter than the rest and it would lower the phylogenetic signal of the analysis. In the phylogenetic tree constructed based on *18S* rDNA (Fig. 2), *cox*1 (Fig. 3), or *rpoB* (Fig. 4) sequences, *S*. *wenzeli* formed an individual clade with *S*. *anasi*, *S*. *albifronsi* and *S*. *rileyi* basal to a group comprising *Sarcocysits* spp. obtained from birds or terrestrial carnivores. Phylogenetic analysis based on ITS1 sequences (Fig. 5) revealed that *S*. *wenzeli* formed an individual clade with *Sarcocysits* sp. isolate Chicken-2016-DF-BR and *S*. *rileyi*, and this clade was within a group comprising *Sarcocysits* spp. obtained from birds or terrestrial carnivores.

## Discussion

*Sarcocystis* spp. in chickens may cause severe myositis [14] and occasionally neurological disease [8, 15]. *Sarcocystis* infection in chickens has been reported in Hungary [1], Bulgaria [2], Russia [16], Papua New Guinea [14], Australia [14], Germany [3], the Czech Republic [17], Azerbaijan [18], China [19], Iran [20] and Brazil [8]. Three species of *Sarcocystis*, *S*. *horvathi*, *S*. *wenzeli* and *S*. *gallinarum*, have been proposed to be responsible for the sarcocysts observed in muscle tissues of chickens. The sarcocysts found in chickens have been divided into two types based on the shape of the bradyzoites. Banana-shaped sarcocysts are considered to be produced by *S*. *horvathi*, described in 1908, which is synonymous with *S*. *gallinarum* whereas lancet-shaped sarcocysts are attributed to *S*. *wenzeli*, described in 1982 [5]. The ultrastructure of the sarcocysts of *S*. *wenzeli* has been described in detail previously [19, 21] and is similar to the type 9k sarcocyst wall classified by Dubey et al. [5]. It is worth noting that morphologically similar sarcocysts have been observed in the lesser snow geese (*Anser caerulescens*) in Saskatchewan, although the species has not been named [22]. The fine structure of the sarcocysts of *S*. *horvathi* and *S*. *gallinarum* is still unclear. In our materials, only the sarcocysts of *S*. *wenzeli* were found and identified, based on the observation of lancet-shaped bradyzoites and the TEM analysis of sarcocysts. The 42.4% (14/33) prevalence rate of *Sarcocystis* identified in chickens was lower than the 94.78% (37/39) prevalence recently surveyed in Iran using the digestive method [20], but was higher than the 8.9% (17/191) prevalence based on microscopic detection reported in China in 2012 [19]. It needs to be stressed that only squash preparation was used to search for mature sarcocysts in tissues of chickens in the present study. Therefore, the prevalence rate of *Sarcocystis* surveyed in the village should be underestimated because of the low sensitivity of the method.

Nucleotide sequence analysis has proven to be a useful tool for delineating or identifying species of *Sarcocystis* from the same or different hosts, and different genetic markers have revealed different levels of intra- or interspecific sequence diversity [6, 7, 12]. There are only one *18S* rDNA sequence, one ITS1 sequence and one *cox*1 sequence of *Sarcocystis* sp. obtained from brains of two chickens in Brazil currently available in GenBank. In the present study, five loci (*18S* rDNA, *28S* rDNA, ITS1, *cox*1 and *rpoB*) from *S*. *wenzeli* were sequenced and analyzed, to the best of our knowledge, for the first time. Among them, *28S* rDNA and *rpoB* constitutes the first records of *Sarcocystis* species in chickens. In our analysis, the sequences of the five loci (*18S* rDNA, *28S* rDNA, ITS1, *cox*1 and *rpoB*) of this parasite presented high intraspecific similarities of 99.8–100%, 99.7–100%, 99.0–99.9%, 100%, and 98.9–100%, respectively. When comparing these sequences with those deposited on GenBank, sequences of *18S* rDNA, ITS1 and *cox*1 of *S*. *wenzeli* shared high similarities with those of *Sarcocystis* sp. isolate Chicken-2016-DF-BR obtained from brains of two chickens, i.e. 99.9–100%, 98.1–98.5%, and 99.3% identity, respectively. Therefore, the unrecognized species of *Sarcocystis* associated with meningoencephalitis in chickens from Brazil in 2020 [8] could be inferred as *S*. *wenzeli* owing to the high similarities of the three loci. The first case of *Sarcocysits*-associated encephalitis in chickens was diagnosed in the USA in 1995 [15], and the species of *Sarcocysits* was not identified because of no sarcocysts observed in brain samples of chickens, similar to the case occurred in Brazil in 2020 [8]. The sequences of the five loci (*18S* rDNA, *28S* rDNA, ITS1, *cox*1 and *rpoB*) of *S*. *wenzeli* exhibited different levels of similarity compared with closely related *Sarcocystis* species, sharing 99.8%, 99.0–99.2%, 89.3–89.7%, 98.5% and 97.5% identity with the corresponding sequences of *S*. *anasi*, and 99.5%, 98.3–98.5%, 78.8–79.0%, 96.4%, and 95.9% identity with those of *S*. *rileyi*. Therefore, ITS1 and *rpoB* appeared to be more suitable to for distinguishing *S*. *wenzeli* from other *Sarcocystis* spp., especially closely related species of *Sarcocystis*, than the *18S* rDNA, *28S* rDNA and *cox*1 loci.

This study also established the phylogenetic relationships between *S*. *wenzeli* and *Sarcocystis* spp. in different hosts based on *18S* rDNA, *cox*1, *rpoB* and ITS1 sequences. The topologies of the trees inferred from these sequences were highly similar and revealed that *S*. *wenzeli* presents a close relationship with *Sarcocystis* sp. isolate Chicken-2016-DF-BR, *S*. *rileyi*, *S*. *albifronsi* and *S*. *anasi*. The later three species utilize geese or ducks as intermediate hosts, and the definitive hosts of *S*. *rileyi* and *S*. *albifronsi* are canines, but that of *S*. *anasi* is still unknown [23, 24]. Based on experimental infection, the definitive hosts of *S*. *wenzeli* were confirmed to be both cats and dogs [3, 25], which is peculiar and differs from the situation for all known *Sarcocystis* spp. found in domestic animals, which use only either cats or dogs as their definitive host. However, *Sarcocystis* sporocysts were not found in the feces of cats fed breast muscle sample from over 2000 chickens from grocery stores in the USA, although the muscle was not examined microscopically for sarcocysts or bradyzoites [26].

## Conclusions

In summary, we found a high prevalence rate of *Sarcocystis* in free-range chickens in China, and only *S*. *wenzeli* was identified based on the cyst ultrastructure. Five loci (*18S* rDNA, ITS1, *28S* rDNA, *cox*1 and *rpoB*) of the parasite were sequenced, analyzed and deposited in GenBank. Based on molecular analysis, *S*. *wenzeli* might be an agent caused neurological disease in chickens. Among these genetic markers, ITS1 and *rpoB* are more suitable for discrimination among closely related *Sarcocystis* species. Phylogenetic analysis revealed that *S*. *wenzeli* shows a close relationship with *Sarcocystis* spp. that use geese and/or ducks as intermediate hosts and canines as definitive hosts.

## Data Availability

The data collected for this study are available from the corresponding author upon request.

## Abbreviations

LM: light microscopy
TEM: transmission electron microscopy
PCR: polymerase chain reaction
*18S* rDNA: *18S* ribosomal DNA
*28S* rDNA: *28S* ribosomal DNA
ITS1: intergenic transcribed spacer region 1
cox1: cytochrome oxidase subunit 1
rpoB: RNA polymerase beta subunit
MP: maximum parsimony
TBR: tree-bisection-regrafting
